# Review of the impact of MALDI-TOF MS in public health and hospital hygiene, 2018

**DOI:** 10.2807/1560-7917.ES.2019.24.4.1800193

**Published:** 2019-01-24

**Authors:** Belén Rodríguez-Sánchez, Emilia Cercenado, Alix T. Coste, Gilbert Greub

**Affiliations:** 1 Department of Clinical Microbiology and Infectious Diseases, Hospital General Universitario Gregorio Marañón, Madrid, Spain; 2 Instituto de Investigación Sanitaria Gregorio Marañón, Madrid, Spain; 3 These authors contributed equally to this work; 4 Department of Medicine, Faculty of Medicine, Universidad Complutense de Madrid, Madrid, Spain; 5 Institute of Microbiology, University Hospital of Lausanne, Lausanne, Switzerland; 6 Infectious Diseases Service, University Hospital of Lausanne, Lausanne, Switzerland

**Keywords:** MALDI-TOF MS, Mass Spectrometry, microorganism identification, antibiotic resistance mechanisms, typing, mycobacteria, antibiotic use, hygiene

## Abstract

**Introduction:**

MALDI-TOF MS represents a new technological era for microbiology laboratories. Improved sample processing and expanded databases have facilitated rapid and direct identification of microorganisms from some clinical samples. Automated analysis of protein spectra from different microbial populations is emerging as a potential tool for epidemiological studies and is expected to impact public health.

**Aim:**

To demonstrate how implementation of MALDI-TOF MS has changed the way microorganisms are identified, how its applications keep increasing and its impact on public health and hospital hygiene.

**Methods:**

A review of the available literature in PubMED, published between 2009 and 2018, was carried out.

**Results:**

Of 9,709 articles retrieved, 108 were included in the review. They show that rapid identification of a growing number of microorganisms using MALDI-TOF MS has allowed for optimisation of patient management through prompt initiation of directed antimicrobial treatment. The diagnosis of Gram-negative bacteraemia directly from blood culture pellets has positively impacted antibiotic streamlining, length of hospital stay and costs per patient. The flexibility of MALDI-TOF MS has encouraged new forms of use, such as detecting antibiotic resistance mechanisms (e.g. carbapenemases), which provides valuable information in a reduced turnaround time. MALDI-TOF MS has also been successfully applied to bacterial typing.

**Conclusions:**

MALDI-TOF MS is a powerful method for protein analysis. The increase in speed of pathogen detection enables improvement of antimicrobial therapy, infection prevention and control measures leading to positive impact on public health. For antibiotic susceptibility testing and bacterial typing, it represents a rapid alternative to time-consuming conventional techniques.

## Introduction

During the past 10 years, Matrix Assisted Laser Desorption Ionization-Time of Flight Mass Spectrometry (MALDI-TOF MS) has changed microbiology routine practice by allowing timely and cost-effective identification of different microorganisms, not only from pure culture but also directly from clinical samples [1-3]. Indeed, faster microbial identification allows for earlier antibiotic streamlining, due to the accurate identification provided for important groups of microorganisms that can be managed with directed antibiotic treatment, as demonstrated when MALDI-TOF MS was applied to bacterial identification directly from blood culture pellets [4,5]. MALDI-TOF MS has also been applied to determine antimicrobial susceptibility patterns, and has produced reliable same-day results; this is a major advantage, as routine antimicrobial susceptibility testing (AST) analyses typically need overnight incubation [6].

MALDI-TOF MS has also emerged as a diagnostic tool for bacterial typing, which could help to detect nosocomial outbreaks, with a putative beneficial impact on disease control and patient safety [7,8]. Hospital hygiene may also benefit from early identification of some emerging and clinically relevant pathogens [9]; in this context, the rapid identification of pathogens, even at the subspecies or serotype level, may positively impact the time until patient isolation and the prompt initiation of the appropriate drug therapy. In some circumstances, such as the recent *Mycobacterium chimaera* outbreak, early identification of atypical mycobacteria would also prove useful to detect such case clusters [10].

Altogether, in this review we aim to demonstrate that MALDI-TOF MS represents a versatile diagnostic technology with great potential to improve the identification of microorganisms and to impact public health by providing important information for optimised antimicrobial stewardship and disease prevention and control.

## Methods

A review of the available scientific literature was carried out. We searched the United States (US) National Institutes of Health’s National Library of Medicine PubMed database for articles published in English between January 2009 and October 2018, using the terms ‘MALDI-TOF’, ‘blood culture’, ‘bloodstream infection’, ‘antibiotic susceptibility testing’, ‘resistance mechanism’, ‘typing’, ‘highly pathogenic microorganisms’, ‘identification’ and ‘diagnosis’.

Reference lists from published articles were also screened to find more literature on the topic. In addition, reports from the European Centre for Disease Prevention and Control (ECDC) were consulted to identify outbreaks and public health issues where MALDI-TOF MS was applied to detect the causing pathogen (https://ecdc.europa.eu/en/threats-and-outbreaks).

The articles identified in the search were screened based on the information included in their titles and abstracts. Studies with a scope other than the application of MALDI-TOF MS on public health and hospital hygiene issues, as well as duplicate studies, were excluded. Case reports, studies acknowledging regional or very local microbiological problems (indicated by a very limited number of samples (n <10) and reviews were also excluded, though their reference lists were checked for related literature. Subsequently, the remaining articles were each assigned to an author for review, according to their area of expertise (including direct application of MALDI-TOF MS on blood cultures (GG), detection of resistance mechanisms (AC, EC), identification of public health-relevant microorganisms and typing with MALDI-TOF MS (BRS, EC).

## Results

### Literature selection

**Figure 1:**
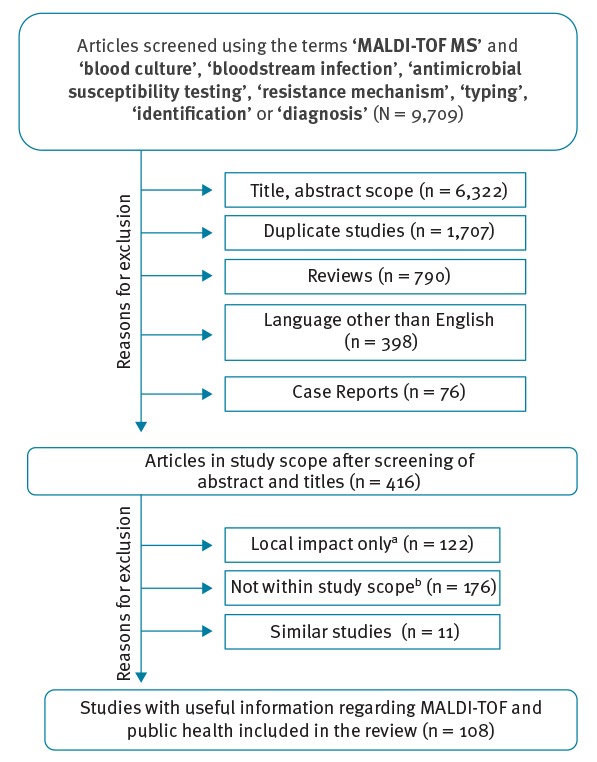
Flowchart of the literature retrieved and retained in review of MALDI-TOF MS use in public health and hospital hygiene, 2018

^a^ Regional or very local microbiological problems indicated by a limited number of samples (n <10).

^b^ MALDI-TOF MS was used as an identification tool, but its performance was not the objective of the study.

A total of 9,709 articles were found using the selected keywords. Based on the information in the titles and abstracts, 6,322 studies were out of scope and were therefore excluded. Among the remaining 3,387 papers, 1,707 appeared in the search results more than once and 790 reviews did not contribute new content because they reproduced results previously obtained by other authors in a different geographical area; these were also excluded. Articles written in languages other than English (n = 398) and 76 case reports referring to a very limited number of samples or patients were excluded as well (Figure 1).

At this stage, the remaining number of references was 416. During a second review, studies acknowledging regional or very local microbiological problems (n = 122) and those where MALDI-TOF MS was used as an identification tool but its performance was not the objective of the study (n = 176) were also excluded. In addition, in order to fall within the maximum number of references for publication, only the most recent articles showing similar design and results were included; all relevant articles are covered here (Figure 1).

Finally, 108 original articles demonstrating proof of concept, as well as a clear impact on microbiology and the microbiology laboratory praxis regarding the application of MALDI-TOF MS, were included in this review.

### Implementation and clinical impact of performing MALDI-TOF MS on blood culture pellets

One of the most impactful uses of MALDI-TOF MS is its ability to identify microorganisms grown in blood cultures [11]. This application has shown to provide reliable identification of possible contaminants and disease-causing pathogens, as well as to reduce turnaround time (TAT) to final identification, since overnight culture on agar media is not necessary [4,12].

Already in 2010, several authors proposed to prepare a bacterial pellet from positive blood cultures in order to fasten pathogen identification [13-15]. Since then, a variety of protocols have been used that reported identification of the aetiological agent of bacteraemia in 70–80% of cases, with accuracy greater than 99% (reviewed in [16]).

In these protocols, sample preparation aims at concentrating the microorganisms present in the blood culture by using differential centrifugation and washing steps. Then, the pellet can be spotted directly on the MALDI target for identification [17] or be submitted to a protein extraction procedure [18]. The use of the SepsiTyper kit (Bruker Daltonics, Billerica, Massachusetts, US) has also been reported for this purpose [19]; its performance was shown to be similar to the direct and protein-extraction methods, but it provided superior results for yeasts identification. These results were supported by several studies [3,20]. In-house methods using different reagents also reported improved identification of yeasts and fungi in blood cultures [21,22]. Croxatto et al. developed an ammonium chloride-based approach to lyse red blood cells and obtain a clean bacterial pellet [23]. A short incubation step right after the blood culture bottle is flagged positive was also tested successfully [24]. The detection of beta-lactamases and carbapenemases using MALDI-TOF MS can also be applied on the obtained pellet. The procedure is detailed further down in this review [6,25].

**Figure 2:**
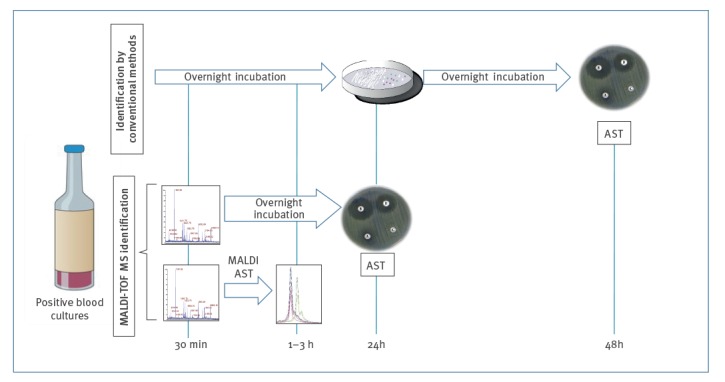
Workflow for the identification of microorganisms from positive blood, review of MALDI-TOF MS use in public health and hospital hygiene, 2018

So far, the drawback of MALDI-TOF MS directly on blood culture detected so far is the inability to identify all bacteria in a polymicrobial infection [17]. It has been overcome by the development of several AST approaches coupled to the identification of the causing pathogen (Figure 2).

AST: antimicrobial susceptibility testing.

On the upper lane, the identification is made from colonies grown on agar plates after a 24-hour incubation; after another 24 hours, AST is performed. In the central lane, the microorganism is identified after 30 minutes using MALDI-TOF MS, but AST is delayed 24 hours. On the bottom lane, optimised MALDI-TOF MS identification and AST is performed within 3.5 hours, completing both tests within one working shift.

The advantage of MALDI-TOF MS over conventional methods is that it offers a reliable identification of the pathogen and AST results can be obtained within one working shift in a rapid and inexpensive manner [26]. The clinical impact of the implementation of MALDI-TOF MS on blood cultures has been measured; in a study by Clerc et al. [5], MALDI-TOF MS allowed the adjustment of antibiotic treatment in 35.1% of the bacteraemia cases analysed. Without considering the centrifugation steps, the cost was calculated to be ca EUR 1.43 per sample tested, whereas the hospital stay was shown to be reduced by ca 2 days, depending on the patient type and the appropriateness of patient management [26,27]. Due to common use of carbapenems for septic shock at their study site, Clerc et al. observed antibiotic streamlining more often than broadening, with routine MALDI-TOF MS applied to blood culture pellets having a clear positive impact on reducing the usage of carbapenems and other broad-spectrum antibiotics [5].

A recent prospective study confirmed that identification of the aetiological agent of bacteraemia by MALDI-TOF MS led to a shorter time to adequate antibiotic treatment [28]. In this study, patients with *ampC*-positive, Gram-negative bacteriaemia rapidly identified by MALDI-TOF MS were optimally treated within 48 hours.

Thus, in several centres the implementation of MALDI-TOF MS for the routine identification of microorganisms directly from blood culture pellets has shown that it may significantly impact the streamlining of antibiotics, with a likely positive impact on the antibiotic resistance rate.

### Identification and typing of epidemiologically relevant pathogens

The high specificity shown by MALDI-TOF MS in different studies encouraged researchers to further analyse the protein spectra obtained for identification of different microorganisms and to attempt comparison between subpopulations.

#### Food-borne pathogens

Discrimination at the subspecies or even serotype level has been researched for different bacterial genera of public health interest. For *Salmonella* spp., the finding of specific peaks that allow genus-, species-, subspecies- and even serotype-level discrimination has been described by Dieckmann et al. [29]. Using a decision tree based on the presence/absence of specific peaks, corresponding mainly to ribosomal proteins, the authors achieved correct identification of the most commonly encountered *S. enterica* subsp. *enterica* serotypes with 100% sensitivity and specificity. More recently, a study using similar peaks as serotype biomarkers and ad hoc software allowed 94% of correct *S. enterica* subsp. *enterica* serotype assignment using a set of 12 species-specific peaks [30]. The authors reported up to 96% correct serotype identification when the software reduced the number of biomarkers used to 10, with no impact on the specificity of the analysis. It is noteworthy that both studies used a whole-cell approach for serotyping, which requires a limited number of reagents and short TAT. The manual process of peak analysis can be more time-consuming and requires trained personnel. However, this requirement can be avoided by implementing specific software for peak analysis. The use of free software such as MALDIQuant [31] allows the simultaneous analysis of many spectra, with the necessity of a trained bioinformatician as the only drawback.

Further important food-borne pathogens that have been successfully subtyped with MALDI-TOF MS are Shiga-toxin producing *Enterobacteriaceae* [9]. The analysis of peak profiles yielded two important biomarkers that allowed correct identification of 103 of 104 *Escherichia coli* O104:H4 isolates from an outbreak that took place in northern Germany [9]. The implementation of MALDI-TOF MS from isolates spotted directly on a MALDI target plate or after a formic acid/acetonitrile extraction renders this methodology very rapid, since the protein spectra can be obtained within minutes.

This approach has also allowed the discrimination of *Listeria monocytogenes*, a pathogen associated with a high mortality rate (20–30%) [32]. Beyond correct species-level identification of *L. monocytogenes* after culture conditions standardisation, the analysis of the protein spectra has allowed the source tracking of *L. monocytogenes* isolates from dairy sources [33] and the correct serotype assignment from clinical samples [34]. In addition, *L. monocytogenes* subtypes can be discriminated using the automated MALDI Biotyper (MBT) subtyping module developed by Bruker Daltonics [35].

#### *Clostridium difficile*

The implementation of MALDI-TOF MS for typing of *Clostridium difficile* has yielded successful results [36]. High molecular weight proteins from 500 isolates were analysed and high correlation with PCR ribotypes (89.0%) was reported. The availability of this easy-to-perform typing method allows rapid and accurate screening of outbreak-related *C. difficile* clones and helps epidemiologists and public health professionals to follow and control putative outbreaks.

#### Respiratory pathogens

Several respiratory pathogens of public health importance have been shown to be reliably identified using MALDI-TOF MS. *Legionella* spp. was identified from environmental samples in two different hospitals in a rapid and reliable manner [37,38].

Attempts to discriminate *Streptococcus pneumoniae* from the members of the *S. mitis* complex have yielded a panel of specific marker peaks that allow species assignment to *S. pneumoniae* isolates and the most common non-pneumococcal species (*S. mitis* and *S. oralis*) [39,40]. Compared with the culture from suspected isolates in the presence of an optochin disk, this MALDI-TOF MS application allows a reduction in TAT and laboratory costs [39].

Another group of important respiratory pathogens are the members of the *Mycobacterium* genus. MALDI-TOF MS cannot differentiate among the species comprising the *Mycobacterium tuberculosis* complex. Nevertheless, its implementation for the identification of non-tuberculous mycobacteria (NTM) has been useful for evaluating the clinical significance of the microorganism recovered by culture of various clinical samples. Around 60 NTM species have been shown to act as opportunistic human pathogens causing pulmonary disease with symptoms similar to tuberculosis lymphadenitis in children associated with *M. avium* and *M. scrofulaceum*, as well as skin diseases and disseminated infections in immunocompromised patients [41,42]. In this scenario, MALDI-TOF MS has shown to provide reliable species-level identification in almost 100% of the cases [43] and the sample processing methods available are easy to apply, require little hands-on time and are widely standardised [44].

However, MALDI-TOF MS applied to NTM grown on liquid medium exhibited a low sensitivity [45]. This drawback has been overcome, however, by some authors using an improved bead-based method for cell disruption. The implementation of this method reduced the TAT up to 2–3 weeks [46]. Closely related NTM species are often identified by MALDI-TOF MS at a complex level. However, Fangous et al. developed an algorithm that allowed the accurate discrimination between three subspecies within the *Mycobacterium abscessus* complex, namely *M. absces*sus subsp. *abscessus*, *M. abscessus* subsp. *massiliense* and *M. abscessus* subsp. *bolletii* [47]. The algorithm was based on the presence/absence of five specific peaks that correlated with the three subspecies. The discrimination of the subspecies within the *M. abscessus* complex was demonstrated as well by Kehrman et al. using principal component analysis [48]. In both cases, the discrimination of the subspecies was accurate and allowed for improved patient management due to the different antibiotic susceptibility pattern of each member of the *M. abscessus* complex. More recently, Pranada et al. have achieved a highly robust and accurate discrimination between *M. intracellulare* and *M. chimaera* by peak analysis [10]. Their approach supports the use of MALDI-TOF MS for the accurate discrimination of NTM isolates associated with heater/cooler devices used for extracorporeal cardiopulmonary support, an important issue in hospital hygiene and infection prevention [49].

#### Biosafety level Risk Group 3 pathogens

Highly pathogenic microorganisms are a major concern for their potential to be used as bioterrorism agents. The identification of Risk Group 3 bacterial pathogens with MALDI-TOF MS was assessed by different groups [50-52]. The authors reportedly showed no identification of these microorganisms when proprietary databases were employed. However, the use of the Security Relevant reference library, developed by Bruker Daltonics, allowed between 52.5–77.0% correct species assignment, although misidentifications with neighbour species were also reported [52]. The rate of correct species assignment reached the totality of the isolates tested only (i) when expanded with in-house libraries and/or (ii) when improved software for spectra analysis were used [51].

Recently, the US Centers for Disease Control and Prevention (CDC) collaborated with Bruker Daltonics in the construction of an expanded library for Risk Group 3 pathogens. This database can be accessed online (https://microbenet.cdc.gov/).

Finally, MALDI-TOF MS was able to identify the emerging pathogen *Candida auris*. The Biotyper updated Research Use Only (RUO) database already contains nine reference spectra from this pathogen, which allowed the discrimination from *C. haemuloni* without using an expanded library (data not shown).

### Antimicrobial susceptibility detection using MALDI-TOF

Even without performing AST, the identification of microorganisms by MALDI-TOF MS impacts antimicrobial stewardship since the common susceptibility pattern of the identified microorganism can be largely deduced. This information can already be partially obtained by direct examination of the sample after performing a Gram staining, but MALDI-TOF MS goes one step further by giving at least the genera of the microorganism. Concerning Gram-negative rods, identification of group 3 *Enterobacterales* or a *Stenotrophomonas *spp. isolate, for example, will modify the antibacterial stewardship. It is the same for Gram-positive cocci and the possibility to distinguish *Enterococcus faecium* from *E. faecalis*, for example.

Considering the continuous emergence of acquired antibiotic/antifungal drug resistance, the need for same-day, full AST results become urgent. From this perspective, several studies have investigated the use of MALDI-TOF MS to perform AST. MALDI-TOF AST assays were first developed to detect specific peaks of resistant strains by peak picking approaches [53-55]. Most of these studies, however, concern detection of drug hydrolysis/modification (reviewed in [56]). Recently, some MALDI-TOF MS assays aimed at detecting drug resistance independently of the biological mechanism, evaluating the growth of a microorganism in the presence of a given drug [57-59].

#### The peak picking approaches

The first MALDI-TOF AST study was performed on *Staphylococcus aureus* to detect meticillin resistance [60]. Comparing the lists of peaks, some peaks specific for meticillin-resistant *S. aureus* (MRSA) and meticillin-susceptible *S. aureus* (MSSA) strains were identified. Further studies were then performed on larger sets of strains and on averaged spectra obtained from several replicates for a given strain. Cluster analysis was performed on the obtained peak list to discriminate MRSA from MSSA strains [61]. Interestingly, some authors of the first study also demonstrated that the cluster analysis result is modified depending on the growth media [62]. In contrast, Bernardo et al. showed that peak profiles were very stable regardless of the growth medium used. However, this study failed to define a clear peak signature for MRSA [63].

Other groups performing peak picking could discriminate between teicoplanin-susceptible vs -resistant staphylococci by analysing peak lists of laboratory-engineered mutant strains [64]. More recently, vancomycin intermediate-resistant *Staphylococcus aureus* (VISA) and vancomycin-susceptible *Staphylococcus aureus* (VSSA) could be discriminated by the identification of 22 relevant peaks using linear regression analysis, followed by a principal component analysis (PCA) on the identified peaks [65]. Once again, the influence of the growth medium on the obtained spectra was documented [65]. In 2018, Asakura et al. [66] further developed the machine learning approach initiated by Mather et al. to discriminate profiles of VISA among MRSA and heterogeneous VISA (hVISA) among MSSA, with 99% sensitivity for both. They also developed an ‘all-in-one’ online software publicly available to analyse in-house spectra [66]. The same approach was used earlier to discriminate *cfiA*-positive and *cfiA*-negative *Bacteroides fragilis* [67].

Since antimicrobial resistance is often due to the production of enzymes modifying the microorganism metabolism or degrading the drug, some MALDI-TOF MS studies developed assays to identify peaks corresponding to such enzymes. Studies were then performed to detect disappearance of peaks corresponding to *E. coli* or *Klebsiella pneumoniae* porins in spectra of strains with high resistance against beta-lactams [53]. This approach allows discrimination between carbapenemase expression and loss of porin expression conjugated with AmpC or extended Spectrum Beta-Lactamase (ESBL). Other groups were able to identify the peak of beta-lactamase at 29,000 m/z in ampicillin-resistant *E. coli* [55]. Concerning the detection of *B. fragilis* resistant to carbapenems, peaks specific to the IS insertion upstream of the *cfiA* gene were determined and a MBT subtyping module from Bruker Daltonics’ was released to detect them [35].

Meticillin resistance in *Staphylococcus* is due to the acquisition of the *mecA* *or mecC* gene. The *mecA* gene is often acquired in parallel to the *psm-mec* gene coding for a toxin. Rhoads et al. specifically detected a peak near 2,415 m/z (± 2.00 m/z) that correlated with meticillin resistance (*mecA* carriage) in a series of consecutive staphylococcal blood culture isolates; this peak was present in 37% of the MRSA and 0% of MSSA strains [68]. Recently, Bruker Daltonic’s MBT subtyping module included the detection of a peak corresponding to the PSM-mec peptide in *Staphylococcus aureus* spectra [35].

#### Detection of drug hydrolysis

The most important outcome of using MALDI-TOF AST so far was the detection of antimicrobial modifications, either quinolones acetylation or beta-lactam ring hydrolysis, leading to mass shift of 43 Da and 18 Da, respectively [56,69,70]. Beta-lactam ring hydrolysis is directly followed by a decarboxylation corresponding to a minus 44 Da shift. Thus, beta-lactamase hydrolysis rather appears as a minus 26 Da shift [71].

In 2011, Sparbier et al. established an interesting table of detected peaks for each type of beta-lactams before and after hydrolysis decarboxylation, in presence or absence of salts [70]. They then correlated the calculated data with measured data on strains incubated for 3 hours with the different drugs. By visual peak analysing, they obtained the same susceptibility and resistance results as routine AST methods. Further studies aimed to detect ESBL *Enterobacterales* through third-generation cephalosporins degradation [70,72]. To quantify the hydrolysis, Jung et al. calculated the logarithm of the hydrolysed/non-hydrolysed peaks. This so-called LogRQ ratio discriminates drug susceptibility with 100% sensitivity and 91.5% specificity [72], even if criteria to interpret the ratio were not clearly defined. In a subsequent study, De Carolis et al. calculated the average intensity of the hydrolysed vs non-hydrolysed peaks, and compared them with the positive and negative control peaks [73]. Both studies investigated the possibility to detect enzyme activity directly in the blood culture pellet and obtained sensitivity and specificity of ca 87% and 98%, respectively.

The majority of the MALDI-TOF AST studies, however, focused on carbapenemases detection, as they represent a challenge for hospital hygiene as an emergent antimicrobial resistance mechanism. Several studies successfully detected carbapenemase-producing bacteria using different carbapenems as substrate, such as ertapenem [74,75], imipenem [76,77] and meropenem [78,79]. However, OXA48 carbapenemase in *Enterobacteriaceae* or imipenemases in *Pseudomonas aeruginosa* remain difficult to detect [80,81]. The addition of a bicarbonate buffer improved hydrolysis by *Enterobacteriaceae* of ertapenem and meropenem, but not imipenem [77,82]. Similarly, addition of zinc ion (Zn^2+^) conserves activity of zinc-dependent *P. aeruginosa* imipenemases [83]. However, Rotova et al. highlighted a slightly better efficacy of meropenem supplemented with sodium dodecyl sulfate (SDS) and bicarbonate to detect *Enterobacteriaceae* and *Pseudomonas* carbapenemases than imipenem plus Zn^2+^ [84].

All these MALDI-TOF MS detections of drug modifications have lead, so far, to the development of the MBT STAR-BL software (Bruker Daltonics) and to one carbapenemase detection kit called MBT STAR-Carba Kit (Bruker Daltonics). Recent studies demonstrated the efficacy of this software with a concomitant identification and detection of ESBL or carbapenemase in around 1.5–5.2 hours, instead of 12–48 hours, with conventional routine protocols [85,86].

#### Detection of global spectra modifications in the presence of a drug

One promising use of MALDI-TOF AST consists of comparing spectra obtained from microorganisms in absence or presence of an antimicrobial agent. This approach was first developed in 2009 to discriminate between fluconazole-susceptible and -resistant *C. albicans* strains [87]. Authors compared spectra of *Candida* cells incubated in increasing concentrations of fluconazole. The minimal profile change concentration (MPCC) was determined as the lower concentration of drug needed to observe modification in the *C. albicans* spectra. Like for classical minimum inhibitory concentration (MIC), breakpoints were defined and then susceptible or resistant phenotypes could be easily determined after a few hours of incubation in fluconazole [87], allowing same-day antifungal susceptibility testing results. De Carolis et al. and Vella et al. further developed spectra comparison, performing a cross correlation index (CCI) matrix with spectra obtained in only three conditions: no drug, breakpoint and high concentration with a reduced 3-hour incubation [88,89]. They also adapted the method to echinocandins [90], other triazoles and other *Candida* species [91]. The overall agreement of the MALDI-TOF AST with the Clinical and Laboratory Standards Institute (CLSI) method ranged from 54–97%, depending on the species and the drug [92].

Comparison of spectra in the presence of a drug was also developed to determine bacterial resistance. It consists of a semiquantitative evaluation of the growth measuring intensity of different peaks in presence/absence of a drug following an internal standard [93]. First assays were performed using meropenem and *Klebsiella* strains [57]. Best results were obtained after 1 hour of incubation, reaching 97.3% sensitivity and 93.5% specificity. Like for the yeast assays described earlier, breakpoints were determined to distinguish susceptible from resistant strains. This approach was enlarged to cefotaxime, piperacillin-tazobactam, ciprofloxacin and gentamicin, other *Enterobacteriaceae* and non-fermenting Gram-negative rods, and it was adapted to blood culture samples [58]. The same methodology was tested for mycobacteria AST and allowed shortening of the TAT to one week for the NTM [59]. An example of such methodology is the MBT-ASTRA kit (MALDI BioTyper Antibiotic Susceptibility Test Rapid Assay, Bruker Daltonics), a promising tool for low-cost, same-day AST results on a wide range of pathogens and drugs [94-97].

### Other applications of MALDI-TOF in public health

The rapid acquisition of protein spectra using MALDI-TOF MS has been implemented as a diagnostic tool for the identification of infection markers. For this purpose, the spectra are usually obtained directly from clinical samples, mainly serum or whole blood [98-100]. This approach could be useful in instances where the pathogen is seldom detected, as is often the case for suspected but unconfirmed fungal infections and for slow-growing microorganisms such as some *Mycobacterium* species. Precisely for these two applications, several authors have recently published interesting data [99-102].

#### Biomarkers for diagnosing fungal pathogens

In the case of fungal infections, Sendid et al. published the first evidence of the presence in serum samples of a disaccharide directly related to experimental invasive candidiasis in a mouse model and also in human sera. They further simplified this methodology and implemented it as routine identification of this biomarker from serum of patients with invasive candidiasis, invasive aspergillosis and mucormycoses [100]. Their results showed that the detection of the disaccharide marker (365 m/z) performed similarly to beta-D-glucan and galactomannan, thus complementing those tests. Although the detection of this biomarker has not been validated yet, its implementation could represent a rapid, inexpensive and easy-to-perform means for detecting invasive infections caused by a wide range of fungal species.

The detection of acute phase proteins with MALDI-TOF MS has also been tested as a marker of antifungal treatment response in a rabbit model of invasive pulmonary aspergillosis [103]. Although these proteins are not specific to fungal infection, their presence in infected rabbits was confirmed, as well as important changes in their expression as a response to antifungal treatment.

#### Biomarkers for diagnosing active and latent *Mycobacterium tuberculosis* infection

Few studies reported the identification of specific plasma biomarkers for latent tuberculosis infection (LTBI), using MALDI-TOF that could differentiate between healthy individuals and those with LTBI. In their study, Zhang et al. (2014) used weak cation exchange magnetic beads (MB-WCX Kit, Bruker Daltonics) to recover plasma proteins even in low concentration. They then acquired spectra of plasma proteins and analysed them with specific algorithms. This combination allowed them to develop a model to discriminate between healthy and LTBI individuals, based on the presence/absence of specific peaks [102]. The same concept was also developed by Sandhu et al., who detected three regions along the protein spectra (around 5.8kDa, 11.5kDa and 21kDa) of plasma samples that also allowed discrimination of healthy individuals from patients with active TB infection and symptomatic LTBI patients with 87–90% accuracy [101]. The advantage of these approaches is that the methodology can be easily standardised, thanks to the use of the commercial kit for protein recovery from plasma. However, the protein ranges analysed by both studies are different and so are the results obtained in both cases. The identification of accurate biomarkers for TB infection would make MALDI-TOF MS a valuable screening tool, though the marker peaks need further confirmation by molecular or serological methods.

#### Biomarkers for diagnosing viral infections

Finally, a similar approach has been applied recently for the identification of a panel of 10 respiratory viruses from infected cell cultures [104]. The authors utilised four commonly used cell lines to establish a background of protein peaks derived from the cell culture and then found specific viral peaks using reference viral strains. The marker peaks were also robustly found in cell cultures infected with viruses from clinical samples. The authors found this methodology to be poorly discriminatory for closely related viruses. The same authors also reported the discrimination of three poliovirus serotypes using MALDI-TOF MS [105].

## Discussion and conclusions

The implementation of MALDI-TOF MS has changed the way many microorganisms of clinical and public health interest are identified. Anaerobic bacterial species, yeasts, mycobacteria and an increasing number of moulds can be reliably identified using this technology. This fact is reflected in the amount of literature about this subject published during the past 10 years (Figure 2). Although only articles referenced in PubMed have been reviewed here, the large number of publications in this database reporting the use of MALDI-TOF MS to rapidly identify a wide range of microorganisms with public health relevance worldwide provides an up-to-date overview of the role of MALDI-TOF MS in this field.

Despite the successful results reported using MALDI-TOF MS and the wide range of scenarios where these findings could be applied, further studies are necessary to standardise the applied procedures and to confirm the reproducibility of the results. In a recent study, the methodology applied for typing was evaluated in different laboratories [106]. Technical and biological replicates were analysed in order to assay the reproducibility of the marker peaks detected in different populations of microorganisms. Their results displayed a reproducibility of technical and biological replicates ranging between 96.8–99.4% and 47.6–94.4%, respectively. Thus, the authors proposed the evaluated technology as a first-line screening tool in outbreak analysis and epidemiological studies. In addition, the use of classifier algorithms and linear support vector machine (SVM) allowed the correct classification of the isolates used for validation. The implementation of these bioinformatics tools, together with standardised procedures and the available software, will turn MALDI-TOF MS into a reliable reference methodology for typing isolates. Free software such as MALDIQuant [31] or proprietary software like FlexAnalysis and ClinProTools (Bruker Daltonics) or Bionumerics (Applied Maths, Sint-Martens-Latem, Belgium) allow automatic analysis of large amounts of protein spectra and facilitates the application of different classifiers for the correct identification of bacterial populations.

Additionally, available databases constructed by MALDI-TOF MS users can now be accessed online for the accurate identification of certain groups of microorganisms (https://microbenet.cdc.gov/) [37,107,108].

Taking into account the great impact of MALDI-TOF MS during the past 10 years, the knowledge that has been acquired during this time and the great flexibility of the technique, we think that its influence in public health will only become bigger in the coming years. Its use for resistance mechanism detection, typing and peak biomarker identification makes MALDI-TOF MS an excellent tool for monitoring the epidemiology of highly resistant or virulent pathogens, for outbreak detection and for screening of isolates within an outbreak, as the rapid acquisition and analysis of the protein spectra would facilitate prompt implementation of isolation measures and the identification of the affected patients. DNA sequencing tests could, therefore, be used as a confirmatory test only, to save time and resources.
